# From Traditional Machine Learning to Fine-Tuning Large Language Models: A Review for Sensors-Based Soil Moisture Forecasting

**DOI:** 10.3390/s25226903

**Published:** 2025-11-12

**Authors:** Md Babul Islam, Antonio Guerrieri, Raffaele Gravina, Declan T. Delaney, Giancarlo Fortino

**Affiliations:** 1 DIMES, University of Calabria, Via P. Bucci, 87036 Rende, CS, Italy; r.gravina@dimes.unical.it (R.G.); g.fortino@unical.it (G.F.); 2 ICAR-CNR, Institute for High Performance Computing and Networking, National Research Council of Italy, Via P. Bucci 8/9C, 87036 Rende, CS, Italy; 3 School of Electrical and Electronic Engineering, University College Dublin (UCD), Dublin 4 Belfield, Ireland; declan.delaney@ucd.ie

**Keywords:** machine learning, deep learning, fine-tuning LLM, hybrid model, soil moisture forecast, smart agriculture, review

## Abstract

Smart Agriculture (SA) combines cutting edge technologies such as the Internet of Things (IoT), Artificial Intelligence (AI), and real-time sensing systems with traditional farming practices to enhance productivity, optimize resource use, and support environmental sustainability. A key aspect of SA is the continuous monitoring of field conditions, particularly Soil Moisture (SM), which plays a crucial role in crop growth and water management. Accurate forecasting of SM allows farmers to make timely irrigation decisions, improve field management, and conserve water. To support this, recent studies have increasingly adopted soil sensors, local weather data, and AI-based data-driven models for SM forecasting. In the literature, most existing review articles lack a structured framework and often overlook recent advancements, including privacy-preserving Federated Learning (FL), Transfer Learning (TL), and the integration of Large Language Models (LLMs). To address this gap, this paper proposes a novel taxonomy for SM forecasting and presents a comprehensive review of existing approaches, including traditional machine learning, deep learning, and hybrid models. Using the PRISMA methodology, we reviewed over 189 papers and selected 68 peer-reviewed studies published between 2017 and 2025. These studies are analyzed based on sensor types, input features, AI techniques, data durations, and evaluation metrics. Six guiding research questions were developed to shape the review and inform the taxonomy. Finally, this work identifies promising research directions, such as the application of TinyML for edge deployment, explainable AI for improved transparency, and privacy-aware model training. This review aims to provide researchers and practitioners with valuable insights for building accurate, scalable, and trustworthy SM forecasting systems to advance SA.

## 1. Introduction

In recent years, agriculture has faced increasing pressure to meet food demands while managing limited resources and adapting to climate change. As a result, there is a rising interest in using digital technologies to improve farming practices. This shift, known as Smart Agriculture (SA), involves the use of tools like Internet of Things (IoT) devices, Artificial Intelligence (AI) models, and cloud-based systems to make farming more data-driven and efficient. These technologies help farmers monitor field conditions; predict environmental changes; and make better decisions with regard to irrigation, fertilization, and harvesting [1,2].

One key area where technology has made a strong impact is in understanding soil conditions. Among these, Soil Moisture (SM) is especially important as it directly affects crop growth, resource planning, and overall farm productivity. In this work, SM refers to the volumetric water content (VWC) of soil, which represents the volume of water per unit volume of soil and is typically expressed in cubic meters per cubic meter (m^3^/m^3^) or as a percentage (%). VWC is a direct measure of the amount of water stored in the soil and is the most common variable estimated by SM sensors such as Frequency Domain Reflectometry (FDR), Time Domain Reflectometry (TDR), and capacitance-based probes.

In contrast, Soil Water Potential (SWP) expressed in kilopascals (kPa) represents the energy required for plants to extract water from the soil, describing the soil’s hydraulic tension rather than its water quantity. While SM (or VWC) is commonly used in irrigation management and soil–water balance studies, SWP is more suitable for plant stress and hydrological modeling. In this work all studies reporting SM or VWC are treated under the same category for comparability, as both variables describe the soil’s water availability from complementary perspectives.

Knowing the SM content helps farmers plan all field events, such as nutrient application, irrigation, and pesticide application, as well as predict lodging events (loss of yield due to the crop falling over) [3]. To monitor and forecast SM, many systems now use sensors that measure real-time conditions such as temperature, humidity, and rainfall. These sensor readings are then combined with AI-based models to predict how SM levels will change in the near future. Although there is a growing body of research on SM forecasting, many existing reviews focus narrowly on sensor hardware or do not fully explore the use of modern AI techniques [4].

This review paper examines how SM forecasting plays an important role in SA. It explores different types of sensors used to collect data and focuses on AI-based models, including Machine Learning (ML), Deep Learning (DL), and hybrid approaches. In addition, it discusses learning strategies and their application in forecasting SM. Along the way, the review highlights current challenges and identifies areas where further research is needed. Finally, it examines new trends such as edge computing and privacy-friendly methods that could improve SM forecasting in the future.

There exist several AI-based solutions for SM forecasting that rely on data collected from a variety of IoT-enabled soil and weather sensors [5,6]. Prior studies have highlighted the importance of these technologies in supporting tasks such as vegetable cultivation [7], smart irrigation scheduling, and early warning systems for drought stress [8]. For example, edge-computing-based systems have been explored for field-level decision-making, while past observations from sensors have enabled time-series predictions using ML and DL models. Several recent reviews [9] have systematically evaluated SM forecasting techniques using the PRISMA methodology, covering important aspects such as sensor types, weather variables, irrigation data integration, feature generation, and evaluation metrics. While this prior work offers a structured view of the domain, it primarily focuses on summarizing existing models and categorizing available sensor technologies. To acknowledge previous research, existing studies are compared with this survey in Table 1. The table highlights differences in scope, methodology, and contributions between this survey and current review articles in the field of AI-driven SM forecasting.

This survey makes several important contributions as follows:

- First, it introduces a clear taxonomy that organizes the technical landscape of SM forecasting, including data sources, algorithms, and learning paradigms.
- Second, it is the first survey to show how modern techniques, such as fine-tuned LLM, cross-farm Transfer Learning (TL), and privacy-preserving Federated Learning (FL), are already being applied to SM forecasting, while also pointing out the gaps that still prevent their use in real-world farming.
- Third, following the PRISMA [10] methodology, this work carefully screened studies published between 2017 and 2025 across multiple databases to ensure quality and reproducibility.
- Finally, the survey frames a set of research questions (RQs) to guide the taxonomy. It not only categorizes existing models but also highlights the key challenges in SM forecasting and outlines promising directions for future research.

**Table 1:** A comparative summary of this survey and existing state-of-the-art reviews in AI-based SM forecasting.

Paper	Prisma Approach	Taxonomy AI/ML/DL	Data Decriptions	Sensor Data	Algorithms	Data Privacy	Tiny ML	LLM	Learning Paradigm	Research Question
[11]	No	Yes	Yes	Yes	Yes	No	No	No	No	No
[12]	No	No	Yes	Yes	Yes	No	No	No	No	No
[9]	Yes	No	Yes	Yes	Yes	No	No	No	No	Yes
[13]	No	No	Yes	Yes	Yes	No	No	No	No	No
[14]	No	No	Yes	Yes	Yes	No	No	No	No	No
[15]	No	Yes	Yes	Yes	Yes	No	No	No	No	No
**This Survey**	**Yes**	**Yes**	**Yes**	**Yes**	**Yes**	**Yes**	**Yes**	**Yes**	**Yes**	**Yes**

This survey paper is organized as follows. Section 2 introduces the review methodology, formulates the key RQs, explains the paper selection process, and presents a novel taxonomy for SM forecasting. Building on this foundation, Section 3 critically examines the selected studies according to the RQs, covering AI-driven models from traditional ML to fine-tuned LLMs, learning paradigms, and the application of SM forecasting in SA. In addition, it discusses the main challenges and outlines future research directions. Finally, Section 4 provides the conclusions of the paper.

## 2. Review Methodology

This section explains the methodology used in this study. It includes the development of a taxonomy to categorize the reviewed works, the process followed for selecting relevant papers, the specific keywords used during the literature search, and the five main research questions guiding the review.

### 2.1. The Goal of the Review

The main objective of this study is to provide a comprehensive review of existing research that focuses on the use of AI models for predicting/forecasting SM. It specifically examines the models deployed in sensor-based environments that collect data from sources such as soil properties, weather conditions, and related variables. In addition, this review aims to highlight how AI-driven forecasting techniques contribute to the advancement of SA by enabling more efficient water use, better crop management, and data-driven decision-making. This type of work is referred to as a survey or review paper. To achieve this, relevant studies were identified through a comprehensive search of journal articles, conference papers, book chapters, and existing reviews directly related to the topic. A clear and systematic process was followed based on the PRISMA approach guidelines [10,16] to ensure that the review is well-organized and trustworthy. This review focuses on papers published in the last 9 years (2017 to 2025) to highlight the most recent progress in the field. All selected papers relate to the use of AI methods to predict SM in agricultural environments.

This work analyzed over 189 research papers and finally selected 68 papers for the survey. A specific focus is placed on papers that use AI models in the context of SM predicting/forecasting towards SA by using ML [4,17,18,19,20,21,22,23,24,25,26,27,28], DL [29,30,31,32,33,34,35,36,37,38,39,40,41,42,43,44,45,46,47,48,49,50,51,52,53,54,55,56,57,58,59,60,61,62,63], LLM [64], hybrid models [65,66,67,68,69,70,71,72,73], and others [64,74,75,76,77]. These selected works included detailed comparisons of algorithms, model performance, and application areas. Details regarding the paper selection process are provided in the following subsection.

To better compare the selected papers and understand recent trends in AI-based SM forecasting, a set of Research Questions (RQs) was created. These questions help us analyze key parts of each study, such as the AI models used, types of sensors deployed, input data features, and the real-world challenges faced during implementation. Moreover, they allow us to explore how fine-tuning LLMs and modern learning paradigms such as FL and TL are increasingly applied in SM forecasting [64,74,77]. The RQ are as follows:

*RQ1:* What types of soil sensors are commonly used for SM forecasting?

*RQ2:* What types of ML models have been used for SM forecasting and analysis?

*RQ3:* How have DL models been applied for SM forecasting, and what advantages do they offer compared to ML approaches?

*RQ4:* Does the hybrid model play a role in SM forecasting, and why is it important with respect to standalone ML and DL models?

*RQ5:* What learning paradigms are most relevant in advancing state-of-the-art SM forecasting?

*RQ6:* What are the challenges and future directions of SM forecasting?

### 2.2. Search Techniques

All relevant research is available through well-known digital libraries such as IEEE Xplore, ACM Digital Library, SpringerLink, ScienceDirect, and Wiley Online Library. Additionally, bibliographic databases and indexing services such as Google Scholar, Scopus, and Web of Science were also used to ensure comprehensive coverage of the literature. ArXiv was consulted as a preprint server to access early-stage research, while ResearchGate was used as a social network to identify related studies and access publications shared by authors. The literature search was carried out between 10 January 2025 and 25 April 2025 using multiple databases and digital libraries.

To enhance the effectiveness of the literature search, keywords related to SM forecasting, prediction, and estimation using AI were applied. The following search string was applied (with slight variations depending on database syntax):

$$\begin{array}{c} (“Soil\;Moisture”\;OR\;“Soil\;Water\;Content”\;OR\;“Soil\;Water”)\;\\ AND\;(“Forecasting”\;OR“Prediction”\;OR\;“Estimation”)\;\\ AND\;(“Machine\;Learning”\;OR\;“Deep\;Learning”\;OR\;“Transfer\;Learning”\;OR\;\\ “Artificial\;Intelligence”\;OR\;“Large\;Language\;Model”) \end{array}$$

More in particular, the following search strings were adapted according to the syntax of each digital library and database:

- **IEEE Xplore:** (“Soil Moisture” OR “Soil Water Content”) AND (“Forecasting” OR “Prediction” OR “Estimation”) AND (“Machine Learning” OR “Deep Learning” OR “Artificial Intelligence”).
- **ScienceDirect/SpringerLink/Wiley Online Library/ACM Digital Library:** TITLE-ABSTRACT-KEYWORDS (“Soil Moisture” OR “Soil Water Content”) AND (“Forecast” OR “Predict”) AND (“Machine Learning” OR “Deep Learning” OR “Artificial Intelligence”).
- **Scopus:** TITLE-ABS-KEY (“Soil Moisture” OR “Soil Water Content”) AND TITLE-ABS-KEY (“Forecast” OR “Predict” OR “Estimate”) AND TITLE-ABS-KEY (“Artificial Intelligence” OR “Machine Learning” OR “Deep Learning”).
- **Web of Science:** TS = (“Soil Moisture” OR “Soil Water Content”) AND TS = (“Forecast” OR “Predict” OR “Estimate”) AND TS = (“Artificial Intelligence” OR “Machine Learning” OR “Deep Learning”).
- **Google Scholar:** “Soil Moisture Forecasting” OR “Soil Moisture Prediction” AND “Artificial Intelligence” OR “Machine Learning” OR “Deep Learning”.
- **Research Gate:** manual keyword search using combinations of *Soil Moisture Forecasting*, *Machine Learning*, *Deep Learning*, *Artificial Intelligence*, and *Sensors*. Publications shared by authors or linked to cited DOIs were manually verified and screened for inclusion.

Three independent reviewers conducted the screening. Disagreements were resolved through discussion and majority agreement. Duplicates, editorials, and non-English papers were excluded. Each included study was recorded in a structured data-extraction sheet capturing author, year, data source, sensor type and depth, model category, input features, data duration, and evaluation metrics.

### 2.3. Eligibility Criteria

The main goal of this review was to explore studies that used soil sensors, weather sensors, or vegetation-related features in SA, focusing on ML, DL, or AI models for SM forecasting. We selected papers that clearly explained how AI or ML was used to support smart farming, improve irrigation planning, or predict/estimate/forecast SM.

We excluded papers during the screening process if they met any of the following conditions:

- The paper only mentions terms such as “Machine Learning,” “Soil Moisture,” or “Deep Learning” in the title, abstract, or keywords, but did not explain or use them in the main content.
- The paper use terms such as “ML” incorrectly or provided unclear or weak explanations.
- The paper was not written in English.
- Articles available only as preprints were excluded from the review to ensure scientific rigor. However, ArXiv was used as a source to identify early-stage research. In cases where a preprint was later published in an updated peer reviewed version, the published version was considered in the survey.

### 2.4. Survey Preference

**Figure 1:**
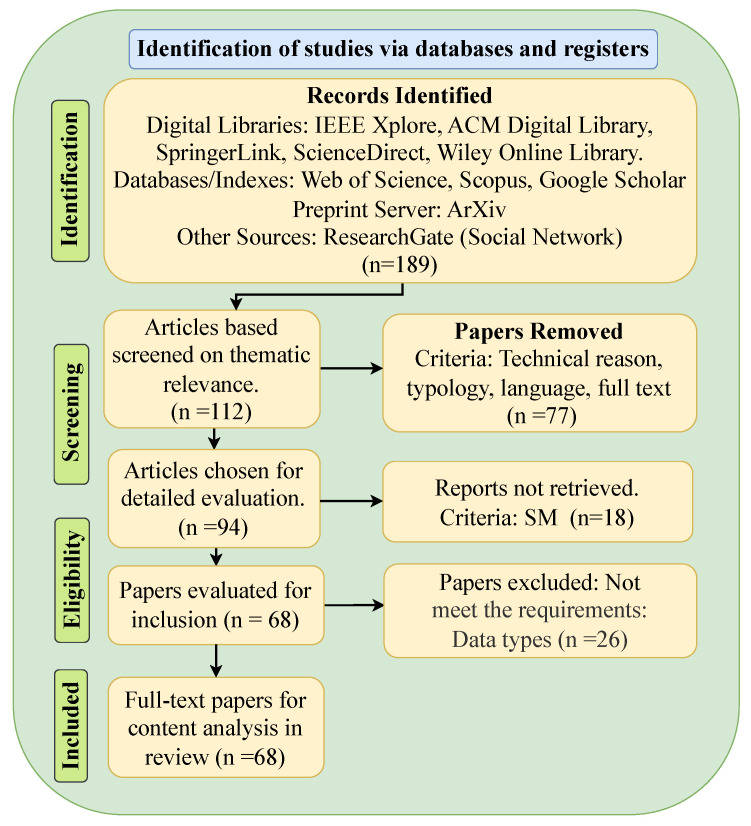
A Prisma-based flowchart of the selection procedure for our survey.

Following the PRISMA 2020 guidelines [10,16], Figure 1 summarizes the study identification and screening workflow. In the identification stage, we retrieved a total of 189 records using a keyword strategy related to SM forecasting, AI models, sensor technologies, and SA. The search covered various types of sources based on keyword-based searches in digital libraries, bibliographic databases/indexing services, a preprint server, and a social network.

To remove non-relevant studies during the Identification stage (see Figure 1), this work applied the following technical filters: (i) excluded publication types such as editorials, short papers, posters, theses/dissertations, brief communications, commentaries, and unpublished works; (ii) excluded papers without full-text availability. After applying these criteria, 77 records were removed, leaving 112 for screening.

In the first screening stage, titles, abstracts, and keywords were independently reviewed by the authors to assess whether the papers met the basic eligibility criteria for SM forecasting. This step excluded 18 records that did not discuss SM forecasting, resulting in 94 articles for detailed evaluation. During the eligibility stage, full texts were examined to verify whether studies focused specifically on SM forecasting using sensor-based data. We excluded 26 papers that relied on other data types (e.g., images or satellite-only sources). Finally, in the included stage, 68 studies met all eligibility criteria and were retained for qualitative synthesis in this review.

### 2.5. A Novel Taxonomy for Soil Moisture Forecasting by Using AI Models

**Figure 2:**
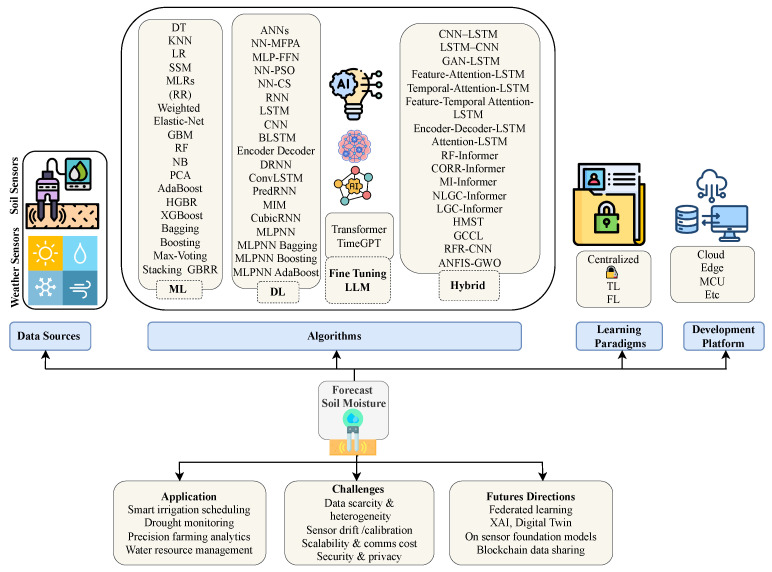
AI-Driven Novel Taxonomy for Soil Moisture Forecasting.

This work proposes a novel AI-driven taxonomy for SM forecasting, as illustrated in Figure 2. The taxonomy provides a comprehensive view of the entire pipeline, encompassing data acquisition, modeling, learning paradigms, platform deployment, and addressing application challenges and future directions. Each component is analyzed in the following subsections.

#### 2.5.1. Data Sources

SM forecasting depends on the integration of diverse and heterogeneous data sources. At the core, in situ soil sensors provide direct measurements of key subsurface variables such as SM, Soil Temperature (ST), and Soil Electrical Conductivity (SEC) [17,18,19,27,28,31,32,33,34,35,36,37,38,39]. These measurements are crucial for capturing the dynamic behavior of moisture in the root zone. In parallel, weather stations contribute important atmospheric variables, such as air temperature, humidity, wind speed, and precipitation, that significantly influence SM through evapotranspiration and infiltration processes [29,30,40,41,42,43,44,45,46,47,48,49,50,51,52,53,54,55,56,57,58,59,60,61,62,63,78].

**Figure 3:**
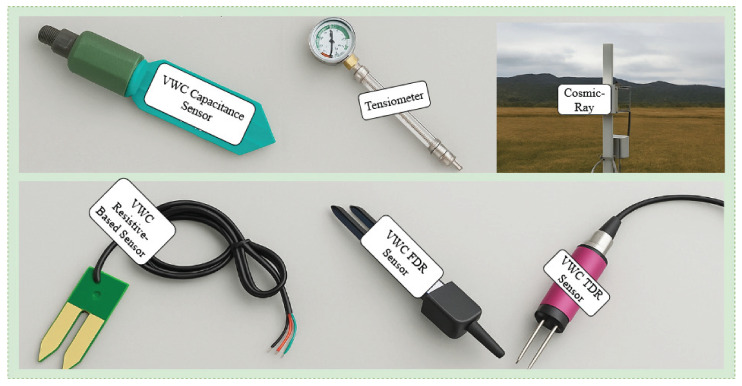
Soil sensors used in SM forecasting include capacitance-based sensors for measuring Volumetric Water Content (VWC), Time Domain Reflectometry (TDR) sensors, Frequency Domain Reflectometry (FDR) sensors, tensiometers, and cosmic ray sensors.

Beyond these, remote sensing technologies and vegetation indices [54] (e.g., NDVI) can also provide additional contextual information when incorporated as numeric features. These sources enable broader spatial coverage and insight into land surface conditions. However, this survey focuses only on numeric sensor-based data and therefore excludes purely image-based approaches.Together, these multi-source datasets serve as essential inputs for training AI models, enabling the development of robust, generalizable, and high-accuracy SM forecasting systems. This data was collected through various SM sensors, which are summarized in Figure 3, and these sensors are described in Section 3.

#### 2.5.2. Algorithms

The core of the proposed taxonomy is grounded in the categorization of AI algorithms into five major classes, each contributing uniquely to the task of SM forecasting.

*Machine learning (ML):* methods represent traditional approaches that rely on engineered features to model patterns in SM data. The common techniques in this category include Decision Trees (DT) [29,44], K-Nearest Neighbors (KNN), Linear Regression (LR), Random Forest (RF) [54], Gradient Boosting Machines (GBMs) [61], XGBoost [61], and various ensemble strategies such as bagging and stacking. These methods are often favored for their interpretability and computational efficiency.

*Deep Learning (DL)*: In contrast, DL approaches leverage neural network architectures capable of learning complex temporal and spatial dependencies from multivariate time series data. This category encompasses models such as Artificial Neural Networks (ANNs) [29,44], Convolutional Neural Networks (CNNs) [62], Recurrent Neural Networks (RNNs) [62], Long Short-Term Memory networks (LSTMs) [53], and advanced variants like Bidirectional LSTM (BiLSTM) [63] and Deep Residual Neural Networks (DRNN) [62].

*Large Language Models (LLMs)*: Moving beyond traditional DL, fine-tuned LLMs have recently gained attention for their potential in time-series forecasting. Models like Transformers and TimeGPT [79] offer powerful sequence modeling capabilities and can be fine-tuned with domain-specific SM data to enhance prediction accuracy.

*Hybrid models*: To capture more intricate patterns and interactions, hybrid models have emerged by integrating multiple architectures. These include combinations such as CNN–LSTM [73], GAN–LSTM, Attention–LSTM, and Encoder–Decoder LSTM models [65]. Additionally, numerous Informer [70]-based variants (e.g., RF–Informer, MI–Informer, LGC–Informer) and attention-enhanced frameworks like GCCL and ANFIS–GWO have demonstrated promising results [70]. This layered taxonomy ensures flexibility and scalability, making it well-suited for the evolving demands of SM forecasting in real-world applications.

#### 2.5.3. Learning Paradigms

Learning paradigms refer to the overall strategy or framework through which a model is trained and updated. The proposed taxonomy integrates modern learning paradigms to enhance model performance and data privacy. Centralized learning involves training models using aggregated data in a central location, but it may raise privacy and scalability concerns. TL [64,74,75,77] is particularly effective under data scarcity situations, as it addresses limited data availability by fine-tuning pre-trained models on local SM data, enabling faster adaptation to new environments. FL [64,74,77] offers a privacy-preserving approach by training models across distributed sources without sharing raw data, making it suitable for sensitive or decentralized soil monitoring systems.

#### 2.5.4. Development Platforms

The development platforms enable scalable and adaptable deployment of SM models. Cloud computing handles complex training tasks [80], while edge devices support real-time predictions near the sensor [81]. For resource-limited environments, MCUs and embedded systems allow lightweight, energy-efficient deployment. This flexibility ensures practical application across diverse field conditions.

#### 2.5.5. Applications

The applications demonstrate the real-world impact of AI-driven SM forecasting. From optimizing irrigation scheduling and enhancing precision agriculture to enabling effective drought monitoring and water resource management, the proposed taxonomy supports diverse, practical use cases that contribute to sustainable agricultural practices and environmental resilience.

#### 2.5.6. Challenges and Future Directions

The taxonomy discusses the key challenges in SM forecasting, including data scarcity, sensor drift, communication costs, and privacy concerns. Future directions focus on FL, explainable AI (XAI), and blockchain to enhance privacy, interpretability, generalization, and data security. This taxonomy offers a structured roadmap for researchers and practitioners in the field of SM forecasting using AI. It bridges classical ML, modern DL, and cutting-edge LLMs while acknowledging deployment concerns and ethical implications. This framework is flexible and extensible to other geospatial and environmental forecasting tasks.

In contrast to earlier taxonomic reviews [9,11,13,14,15], which primarily categorized SM forecasting models into ML, DL, or hybrid groups, the present taxonomy broadens the scope by explicitly incorporating LLMs such as TimeGPT; integrating modern learning paradigms including FL, TL, and TinyML; and linking these to deployment levels (Cloud, Edge, MCU, TinyML). This integrated structure highlights the transition from traditional data driven approaches to privacy aware and resource efficient AI architectures. Therefore, the novelty of the taxonomy lies in unifying algorithmic classes with deployment environments and emerging paradigms, rather than repeating prior categorizations.

## 3. Literature Review

This section begins by analyzing the set of papers selected for the study. As part of the review, we examine the data sources, types of sensors, and forecasting algorithms used across studies. We also explore the practical applications of these models, discussing their strengths, limitations, and relevance to real-world deployment. It then addresses the research questions introduced in the Section 2.1.

### 3.1. Traditional Machine Learning in Soil Moisture Forecasting

This subsection addresses both RQ1 and RQ2 by examining traditional ML models for SM forecasting, as well as the range of sensors utilized in SA. To give a structured overview of the literature, Table 2 summarizes the main case studies, highlighting the ML algorithms, sensor types and depths, data features, study location, data collection durations, and evaluation metrics used.

In the literature, researchers have employed a variety of ML algorithms across diverse geographical regions, soil types, and environmental conditions. Among the most commonly used models for SM forecasting, there are SVM [17,19,21,22], along with DT and KNN [4]. RF has also been widely applied due to its robustness in handling nonlinear relationships and mixed input features [20,25,27].

In addition to single models, several studies explored ensemble learning techniques, which combine predictions from multiple base learners to improve accuracy and reduce overfitting. These include Gradient Boosting Machines (GBM), XGBoost, Bagging, Stacking, Max Voting, and Histogram-Based Gradient Boosting (HBGB) [23,24,26]. Ensemble methods consistently outperformed single learners in environments with high variability in soil conditions, weather patterns, and sensor configurations, highlighting their potential for more generalizable SM forecasting systems.

Other studies have employed a range of regression-based models, including LLR, Multiple Linear Regression (MLRs), Weighted LR, Ridge Regression (RR), Lasso Regression, and Elastic Net [17,18,26]. These models are computationally efficient and highly interpretable, making them suitable for quick implementation and environments with limited resources. However, a key limitation is their inability to effectively model nonlinear or complex temporal relationships, which are common in SM dynamics.

In a few instances, Naive Bayes classifiers were applied, especially when probabilistic predictions were needed, though their use in SM forecasting remains relatively limited [19]. To address the challenge of high-dimensional input data, some studies incorporated dimensionality reduction techniques, such as Principal Component Analysis (PCA), to simplify the feature space and reduce noise, ultimately improving model performance and training stability [19,26].

Referring to RQ1, a wide variety of soil sensors have been used in conjunction with ML models to forecast SM, as summarized in Table 2. These sensors represent a mix of technologies selected based on field conditions, data requirements, and deployment constraints. Frequency Domain Reflectometry (FDR) is one of the most commonly used methods due to its reliable accuracy, low maintenance, and ease of installation [4,19,20,26]. Capacitance-based sensors, as used in [18], are also popular for their cost-effectiveness and ability to provide multi-depth readings. Resistive sensors, adopted in studies such as [17,24], offer an inexpensive option, although they can be less reliable in soils with high salinity or temperature variability.

Despite their importance, several studies do not clearly report the type of sensor used, and these have been labeled “NR” (not reported) in this analysis [21,22,23,25]. The sensor depths varied considerably across studies, with commonly reported depths including 5, 10, 25, 30, 50, and 100 cm. These depths span from shallow surface measurements to deep root-zone monitoring, which is critical for understanding plant water uptake and subsurface SM dynamics. The variation in depth matters significantly, as SM conditions often change with soil structure, irrigation strategies, and vegetation type. A number of studies did not report the depth of the sensor completely or were not applicable, which is defined as (-) in the table, which poses challenges for reproducibility, cross-site comparison, and generalization of forecasting models.

In most studies, input features were derived from soil and weather sensor data, such as SM, ST, Air Temperature (ATT), Relative Humidity or Humidity (HU), Rainfall (RL), Solar Radiation (SR), and others. Some works also included additional environmental and agricultural variables like Wind Speed (WS), vegetation type, leaf wetness, elevation, slope, Crop Evapotranspiration (CE), and SEC [4,20,26]. The studies were conducted in a wide range of geographic regions, including India, China, Romania, Netherlands, Turkey, Taiwan, Bangladesh, and the United States, which introduces variability in soil properties, climate patterns, and agricultural practices. While this diversity demonstrates the adaptability of ML methods, it also makes cross-region model transferability a key challenge.

Model performance across the reviewed studies was primarily evaluated using standard error-based metrics, including Mean Squared Error (MSE), Root Mean Squared Error (RMSE), Mean Absolute Error (MAE), and the Coefficient of Determination (R^2^) [4,20,27]. These metrics provide a quantitative basis for assessing model accuracy and reliability. In addition, several studies incorporated complementary metrics such as Mean Absolute Percentage Error (MAPE), Bias, Unbiased RMSE, and the Nash–Sutcliffe Efficiency (NSE) to offer deeper insights into forecasting performance under different conditions [20,26]. The use of these diverse metrics supports more robust and consistent comparisons across varying datasets, sensor configurations, modeling approaches, and geographical regions.

One of the major limitations of traditional ML models is their reduced ability to capture temporal dependencies in time-series data, which is critical for SM prediction. Since SM is influenced by previous rainfall, evaporation, and irrigation, simple models such as LR or DT may miss important lag-based patterns. To address this, some researchers introduced engineered time-lag features or adopted ensemble and optimization strategies to strengthen the models’ temporal awareness [26].

The duration of study in datasets varied significantly. Some studies used very short-term datasets, such as one week [17] or 40 days [24], while others had long-term datasets up to 39 years [25]. This variability affects model robustness, especially in seasonal or climate-sensitive environments. Cross-validation was used in several studies to improve reliability, but in some cases, details about data splitting, training, and validation were not clearly explained.

In summary, traditional ML models have played an important role in early efforts at SM forecasting, especially where datasets were limited and interpretability was deemed important. Their inability to fully capture time-based patterns, along with inconsistencies in sensor data reporting, limits their effectiveness in more complex or real-time applications. These challenges have encouraged researchers to explore more advanced approaches, such as deep DL and hybrid models, which will be discussed further in the next sections of this review.

### 3.2. Deep Learning Models in Soil Moisture Forecasting

This subsection addresses RQ3 by examining how DL models have been applied for SM forecasting. In contrast, ML methods, which often require manual feature engineering and have limited ability to capture long-term dependencies, DL architectures can automatically learn nonlinear patterns from multivariate time-series data. In addition, DL models are based on data collected from soil and weather sensors, and as such, also relate to RQ. To provide an overview of these applications, Table 3 summarizes the key studies employing DL techniques, including the models used, sensor types and depths, data features, study durations/location, and evaluation metrics.

As shown in Table 3, various DL models have been applied, thus far in the literature for SM forecasting, including ANNs, CNNs, RNNs, LSTM, and Bidirectional LSTM (BiLSTM). Recent models, such as ConvLSTM, PredRNN, and CubicRNN, have also been introduced. These DL models work well with time-series data as they can learn complex patterns, use many input features, and build relationships of how values change over time. As a result, DL approaches have gained increasing popularity for improving forecasting accuracy, scalability, and automation, positioning them as a key component in the development of intelligent SM systems within SA frameworks.

Among all DL models, LSTM was the most widely adopted across the studies [38,41,53,78]. This is primarily due to its effectiveness in capturing long-term temporal dependencies in time-series SM data. Additionally, ANNs remained popular, particularly in earlier studies [29,30,55], and were frequently used in comparative evaluations between ML and DL models [48,52]. A number of papers also included traditional statistical models like ARIMA and SARIMA as baselines for benchmarking DL performance [46,56,60]. A few studies further expanded the modeling space by implementing Deep Recurrent Neural Networks (DRNNs) and other advanced time-series networks for better handling of nonlinear temporal dynamics, although these remain less frequently explored. For example, Ref. [62] applied DRNN and a wide set of DL architectures, including CNNs and LSTMs, in ensemble settings for SM prediction. Some researchers utilized Nonlinear Autoregressive Networks with Exogenous Inputs (NARs/NARXs) [47,62] to model feedback systems and time-lagged dependencies. These models were especially valuable in scenarios with cyclical soil-water behavior, such as irrigation feedback loops. In terms of fast-training alternatives, Extreme Learning Machines (ELMs) [57] were explored for their low computational cost and fast convergence in [57]. To improve generalization and reduce overfitting, several papers adopted ensemble DL approaches, particularly Multi-Layer Perceptron Neural Networks (MLPNNs) combined with Bagging, Boosting, and AdaBoost strategies. These ensemble techniques, seen in studies such as those by [57,62], demonstrated improved robustness across spatial and temporal conditions.

A variety of sensor types were employed to collect SM data using DL models. *Referring to RQ1*, DL models generally use similar types of sensors as traditional ML models, such as capacitance and FDR. Some DL studies also used capacitance-based sensors (e.g., TEROS 12), which detect changes in the soil’s dielectric properties and are valued for their affordability and ease of deployment. More precise measurements were obtained through the use of Time Domain Reflectometry (TDR) [53] and the gravimetric method, the latter being a laboratory standard for SM estimation. Additionally, cosmic ray [59] sensors were used in certain DL models to provide non-invasive, spatially averaged SM measurements. These sensors detect neutrons reflected from hydrogen atoms in the soil, making them suitable for estimating moisture over larger areas and at greater depths. Some studies used EC-TM [62] probes, which can measure both SEC and SM at the same time. This is useful for understanding salinity levels and nutrient conditions in the soil. Weather stations were also commonly used to collect environmental variables such as temperature, HU, and RL, which are important inputs for SM forecasting. In addition, a few recent studies used satellite-based remote sensing data such as ERA5 [63], which provides wider spatial coverage for surface-level SM estimation, although it offers limited information about deeper soil layers. Sensor depth varied across studies, ranging from shallow levels (5–10 cm) to deeper layers (50–100 cm), depending on the root zones being monitored. These depth differences are important as SM changes with depth due to root activity, soil type, evaporation, and irrigation methods. Several papers did not clearly mention the sensor type or depth; however reduces reproducibility and limits the ability to generalize the models to other locations.

Most studies relied on sensor-based environmental data such as SM, ST, ATT, HU, RL, WS, and SR. Some also included more advanced features like SEC, leaf wetness, UV index, Vapor Pressure Deficit (VPD), and vegetation indices [40,58]. A few studies used remote sensing or cosmic ray sensors to capture SM over larger areas and with higher spatial variation [30,59]. These studies were conducted in various countries. It shows that DL models can adapt to diverse conditions, but also suggests that region-specific tuning is needed for the best results.

In terms of evaluation, most papers adopted standard metrics such as RMSE, MAE, MAPE, NSE and R^2^ [33,42,78]. Some studies also included additional metrics such as unbiased RMSE and Graphical Performance Index (GPI) to offer a broader view of model reliability and generalization [27,61].

In the literature, a major strength of DL models is their ability to work with different types of data, regardless of the length or resolution. Some studies used short-term data, such as 8-day experiments [56], while others used long-term climate records, even decades of ERA5 data [63]. When only limited data are available, however, there is a higher risk of overfitting or underfitting, and model choice and tuning become particularly important. Different models performed better depending on the data. LSTM and RNN models performed well with longer periods, as they can learn patterns over time. In contrast, CNN and ConvLSTM models were suitable for temporal and spatial data, particularly when using grid-based or multi-depth sensor data.

Despite their strengths, DL models also face some challenges. Generally, a key issue is the need for large and high-quality labeled data. In many regions, data is limited or inconsistent, there may be missing values, different sensor types, or measurements only at shallow depths, which can make the models less robust. To overcome this, some studies used methods like Principal Component Analysis (PCA) [40] to reduce data complexity, or feature selection techniques such as Boruta [59] to keep only the most important inputs. Others improved performance by combining data from multiple depths in a single model. A few studies also used data augmentation and resampling to deal with uneven or imbalanced measurements across time and depth.

In summary, DL models have improved SM forecasting by providing more accurate, flexible, and scalable solutions than traditional ML models. Their ability to learn complex patterns makes them a good fit for smart farming. Issues like limited data, however, privacy, and deployment still need attention.

The next paragraph introduces fine-tuning LLMs, an emerging direction that extends DL models by leveraging transformer-based foundation architectures for soil monitoring.

#### Fine-Tuned LLM for Soil Moisture Forecasting

Recent research has also begun to explore the use of foundation models and LLMs for time-series forecasting in agriculture. Although originally designed for natural language processing, LLMs such as GPT and BERT have been successfully adapted to domains including computer vision [82], healthcare [83,84], finance [85], agriculture [86] and time-series forecasting [87]. To learn sequential patterns, these models use transformer-based architectures with self-attention mechanisms and temporal encodings. Fine-tuning in this context means adapting a pre-trained model to a specific dataset by updating its weights using a smaller amount of labeled data. This technique offers two main advantages, as described in the following:

- It reduces the need for large training datasets, which is often a challenge in domains like agriculture.
- It lowers the computational cost compared to training models from scratch.

In the context of SM forecasting, this research direction is still in its early stages. A pioneering study by Deforce et al. [64] applied a fine-tuned version of TimeGPT to predict soil water potential, a variable closely linked to SM and field water status. Their evaluation considered three scenarios: zero-shot (no retraining); fine-tuning using only past soil data; and fine-tuning with additional exogenous variables such as rainfall, ST, and evapotranspiration. In the context of SM modeling, these factors are often treated as exogenous inputs since they strongly influence SM dynamics but are not direct measurements of SM itself. Results showed that the fine-tuned model without these additional variables still performed competitively, surpassing traditional ML and several DL baselines. Interestingly, including exogenous variables sometimes reduced performance, likely due to mismatches with the pre-training distribution. This finding demonstrates both the adaptability and the sensitivity of foundation models when applied to SM forecasting. The significance of this work lies in showing that foundation models can be adapted to SM forecasting tasks with minimal fine-tuning, achieving strong results even when only past target data are available. This approach highlights the potential for data-efficient and scalable forecasting, particularly valuable in regions with limited sensor coverage.

Building on this foundation, researchers have also explored the role of LLMs in making forecasts more explainable [88]. For example, a framework combining SM prediction models with an LLM and a domain ontology was proposed to generate human-readable explanations, thereby increasing farmer trust and decision-making ability without sacrificing predictive performance [88].

On a broader scale, EnvGPT, an LLM fine-tuned on environmental corpora, demonstrated state-of-the-art performance on reasoning and QA tasks in water resources and soil management, highlighting how domain-specific fine-tuning can adapt LLMs for environmental decision support, though direct SM forecasting tasks remain underexplored [89].

Table 4 summarizes the works in the literature in which LLM-based models are used for soil moisture forecasting. While the application of LLMs to SM forecasting remains limited, existing experiments demonstrate their potential for both accurate prediction and improved interpretability. Early studies show that foundation models such as TimeGPT can outperform traditional approaches with minimal fine-tuning, and ontology-driven frameworks and domain-specific models like EnvGPT highlight the broader role of LLMs in environmental decision support. These developments establish a solid foundation on which future research can build.

It is important to note that trends in recent studies indicate that LLM fine-tuning often involves a small portion of the pre-training dataset and is completed within several GPU hours, depending on hardware configuration and model scale. In contrast, traditional AI models such as LSTM generally train faster and consume less energy but lack the generalization and transfer capabilities of foundation models. Since exact computational costs and fine-tuning parameters vary widely across studies, this review emphasizes methodological diversity and future research potential rather than direct numerical benchmarking.

### 3.3. Hybrid Models for Soil Moisture Forecasting

This subsection addresses RQ4 by analyzing how hybrid models have been applied for SM forecasting. Hybrid models are designed to integrate the complementary strengths of different approaches. By combining multiple learning strategies within a single framework, they can capture both temporal and spatial patterns more effectively, improve robustness against data variability, and enhance forecasting accuracy compared to standalone ML or DL methods. These models also provide greater flexibility for handling heterogeneous data sources and long-range dependencies. Since all hybrid frameworks are ultimately trained on data collected from soil and weather sensors, this subsection also relates to RQ1. To provide a structured overview of these approaches, Table 5 summarizes the main hybrid studies, including the models used, sensor types and depths, data features, study durations, and evaluation metrics. As shown in the table, hybrid frameworks have been widely explored in the literature so far. These approaches often outperform standalone ML or DL methods by better capturing spatiotemporal variability, reducing overfitting, and improving generalization across diverse environments.

For example, Ref. [67] combined LSTM and attention mechanisms with genetic algorithms (GA) to optimize feature selection, improving the predictive performance of time-series data with long past records. Similarly, Ref. [71] proposed a Hybrid Metablend Stacking Technique (HMST) by blending models like RF, SVM, and MLP into a stacked ensemble architecture. Several works also applied hybrid approaches, combining LSTM with other architectures such as CNNs [66], encoder–decoder structures [90]. In addition, some studies have explored more specialized or hybrid approaches. For example, Ref. [72] tested models such as Adaptive Neuro-Fuzzy Inference Systems (ANFIS), ANFIS combined with the Grey Wolf Optimizer (ANFIS-GWO), and a hybrid RF Regressor with CNN (RFR-CNN). They also evaluated other methods like Case-Based Reasoning (CBR), Support Model Regression (SMR), and Boosted RF (BRF). These models were typically applied when working with multiple heterogeneous data sources or combining soil, weather, and remote sensing variables, but they are less commonly used across the broader SM forecasting literature.

Many studies focused on transformers, which form the architectural backbone of many modern LLMs. It has also been adapted for time series forecasting in SM studies, but often in hybrid configurations rather than single models. Advanced transformer-based models were hybridized in novel ways to improve long-range sequence modeling and multi-variable fusion. For example, Ref. [70] evaluated long-range dependencies in surface SM across globally distributed sites by incorporating multiple Informer model variants—including *Causal–Informer*, CORR–Informer, and *Autoformer* in combination with LSTM and RF. Similarly, Ref. [69] fused *Informer* with *PCA* and *Variational Mode Decomposition* to extract key patterns and reduce noise from long-term time-series data before prediction. These hybrid approaches illustrate how transformer models can be adapted and enhanced to fit domain-specific forecasting needs, even before the emergence of fine-tuned foundation models like TimeGPT.

Graph-based learning also emerged in hybrid designs. For example, Ref. [73] proposed a GCCL model, which integrates graph convolutional networks with ConvLSTM to handle both spatial and temporal relationships in SM prediction. Similarly, Ref. [65] presented a highly diverse model comparison including GAN-LSTM, CNN-LSTM, temporal attention modules, and 1D-CNN, offering deep feature extraction, time-series modeling, and attention mechanisms within unified pipelines.

Some studies used global environmental datasets like *FLUXNET*, *NASA POWER*, and *ISMN* in their hybrid models. *FLUXNET* is a global network of micrometeorological towers that measure CO_2_, water, and energy fluxes between land and atmosphere, offering rich environmental context. *NASA POWER* (Prediction of Worldwide Energy Resources) provides meteorological and solar radiation data tailored for agriculture and environmental modeling. *ISMN* (International SM Network) aggregates in situ SM data from multiple global networks, supporting consistent multi-site analysis. The input features used in these hybrid models were similar to those in traditional ML and DL models. In some cases, extra variables were included, such as *CO_2_*, *soil heat flux*, and *latent/sensible heat flux*. The models used data from different sensor types, including *capacitance-based sensors*, *FDR*, *remote sensing sources* like SMAP L4 and ERA5-Land, and ground station networks. On the other hand, sensor depths in these studies ranged from *shallow surface layers (5–10 cm)* to *deep profiles (up to 1 m)*, enabling root zone-level monitoring.

The majority of evaluation metrics used are the same as ML and DL models. Some studies also incorporated advanced evaluation tools such as *Pearson correlation (CORR)*, *SHAP values* for feature importance, and *t-SNE visualizations* for model interpretability [65].

In summary, hybrid models offer a flexible and powerful approach to SM forecasting by combining different learning strategies. These models are particularly valuable when handling *multivariate, long-term, or globally distributed datasets* and often outperform standalone models. As research progresses, hybrid methods may provide a practical balance between predictive accuracy and interpretability, serving as effective tools for decision support in SA.

Several recent studies have shown promising results but still have limitations. For example, the papers [73,73] use only time-based graph links, while [70] relies on Granger causality, which may not reflect true physical causes. The paper [65] does not include satellite data (e.g. NDVI), static soil characteristics (e.g. texture, topography), or tests of advanced transformer- or physics-based models. Future work should include multi-depth data, satellite and static features, causal or physics-informed methods, and finer time resolutions for better accuracy and generalization.

The reviewed studies collectively illustrate the progression of modeling strategies for SM forecasting rather than providing directly comparable quantitative outcomes. While some works report improved accuracy using deep and hybrid architectures, the reported metrics (e.g., RMSE, MAE, $R^{2}$) are not standardized across datasets, depths, or time horizons. Therefore, instead of emphasizing absolute values, this review focuses on identifying methodological and architectural trends.

In general, traditional ML methods such as RF or SVMs are applied to surface-level measurements with limited feature sets, whereas DL models (e.g., LSTM, CNN) increasingly integrate multi depth soil data and meteorological features. Hybrid and transformer-based approaches extend this trend by coupling spatial-temporal learning with feature attention and multi-depth forecasting capability. However, challenges such as inconsistent sensor configurations, heterogeneous feature availability, and varying temporal resolutions continue to limit model generalization. Future work should emphasize standardized data formats and benchmark protocols to enable meaningful performance comparisons.

### 3.4. Learning Paradigm and Development Platform

In SM forecasting, the most common learning paradigm is centralized learning, where data are collected and stored in a single location, and the model is trained using that complete dataset. Although this approach is straightforward and widely used, it becomes less effective when data are distributed across multiple locations or when privacy, computation, or communication constraints prevent centralized data collection. In such situations, decentralized learning is preferred. Relying solely on centralized learning often results in two key problems:

- *Data scarcity*: New farms often have only a few days or weeks of data available.
- *Privacy concerns:* Many farm managers are not willing to share raw data due to security or ownership issues.

To address these limitations, alternative learning paradigms have emerged, such as TL and FL. TL involves taking a pre-trained model, typically trained on a large, generic dataset, and fine tuning it on a smaller, task-specific dataset. This approach is useful in scenarios where labeled data is scarce or expensive to obtain, which is often the case in SM monitoring across different regions or sensor setups. FL enables decentralized model training across multiple devices or data sources without sharing raw data. Each local node trains a model on its data and shares only model updates (e.g., gradients) with a central server, preserving data privacy.

In the SM literature, TL allows models to learn from one domain and then be adapted to a new, similar task using much less local data. For example, the paper [74] showed that a ConvLSTM model pre-trained on global SMAP satellite and ERA-5 weather data could be fine-tuned using just 2% of local sensor data. This significantly improved accuracy, reducing the error by 15%, and achieving an R^2^ of 0.91. Another study built on the same idea; the paper [75] applied TL to move a CNN model from one climate region (alpine grasslands) to another (páramo highlands) and reduced MAE by 0.013. The newest twist comes from foundation models such as [64] using TimeGPT, a transformer trained in 100 million public time series, and achieving LSTM-beating skill after fewer than 500 local gradient steps. Collectively, these papers confirm that TL is a practical on-ramp whenever data are scarce or fast deployment is required. Together, these studies show that TL is especially useful when local data is limited or when quick deployment is needed in new fields or regions.

In a real-world trial, the paper [76] implemented FL in a rice field using 14 edge devices. The locally trained model (a small MLP) achieved nearly the same performance as a cloud-based model, with only a 9% drop in accuracy, while keeping all raw data on the farm. A follow-up study [77] ran the same FL model on a tiny 32-bit microcontroller using the FedProx algorithm and showed potential for reducing water use by 25%. Although FL is promising, a recent [91] survey noted that many agricultural FL studies do not report important details such as differential privacy budgets or how well the models scale beyond small networks of a dozen devices. From the reviewed papers, learning paradigms such as TL and FL emerge as the most commonly applied approaches in SM forecasting. The answer to *RQ5* can be summarized as follows:

1. TL helps when data is limited and boosts model performance with minimal training.

2. FL enables private and distributed collaboration, which is critical for real-world agricultural deployments.

Table 6 summarizes all the works in terms of learning paradigms, AI models, sensor types and depths, data duration with location, and evaluation metrics.

**Figure 4:**
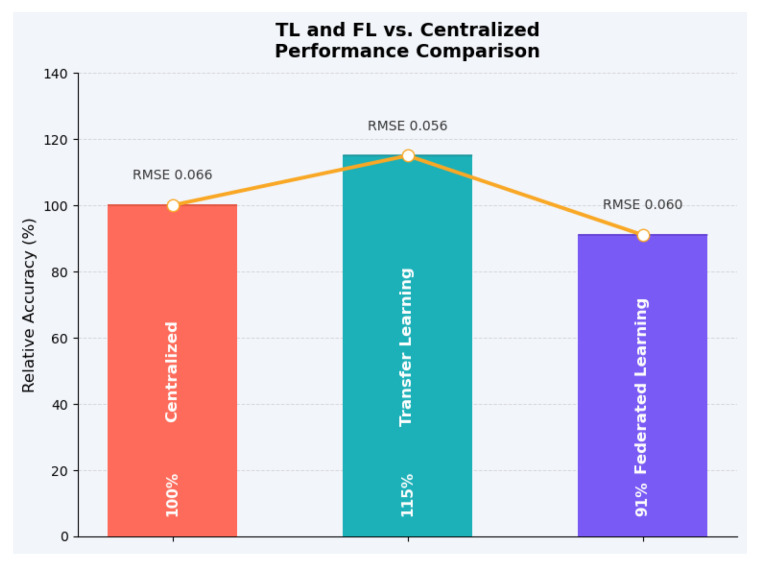
Quantitative comparison of Transfer Learning (TL) and Federated Learning (FL) relative to centralized training for SM forecasting (data summarized from [64,74,75,77]). RMSE values are taken from the literature, while relative accuracy values are normalized to the centralized baseline (100%) to indicate proportional improvement or retention.

Empirical results summarized from [64,74,75,77] indicate that *Transfer Learning (TL)* reduces the RMSE by approximately 15% (from 0.066 to 0.056), while *Federated Learning (FL)* achieves an RMSE of approximately 0.060, with only a 9% decrease in accuracy compared with centralized training. To provide a clear comparison, these values were normalized relative to the centralized model (baseline = 100%). Under this normalization, TL corresponds to approximately 115% relative accuracy (a 15% improvement), whereas FL corresponds to approximately 91% relative accuracy (a 9% retention loss). Figure 4 summarizes these quantitative relationships, highlighting TL’s generalization benefit and FL’s privacy advantage in distributed learning environments.

### 3.5. Soil Moisture Applications and Deployment Contexts

Most of the reviewed studies show that SM prediction models are very helpful in agriculture. They support smart irrigation, precision farming, and efficient water use. These models help farmers decide when and how much to irrigate, improving both efficiency and crop yield. Some models are designed for specific crops, such as soybeans or tea [22,53,92], while others are suitable for general farming [36]. Many ML and DL models are also used in smart farming systems with IoT devices. Overall, SM forecasting supports the following:

- Precision irrigation;
- Crop health monitoring;
- Water resource management;
- Climate and drought analysis.

These applications are typically executed in two main deployment contexts. The first involves IoT-based smart farms, where in-field sensors collect real-time data to guide irrigation, and models are often embedded in smart controllers to automate water delivery [18,24,38]. Some recent works, such as [77], have further explored edge-centric systems, enabling real-time learning and inference directly on local devices without reliance on cloud infrastructure. The second context is regional or remote environments, which rely on weather or satellite data to predict SM levels over larger areas. These are particularly useful for national water planning, drought detection, and agricultural forecasting at scale [20]. However, most studies in this category focus more on model accuracy than on explicit deployment details such as edge or cloud integration.

### 3.6. Overall Challenges and Future Research Direction

This section, referring to RQ6, highlights the main challenges in SM forecasting, including data scarcity and heterogeneity, lack of standardized benchmarks, model interpretability, and deployment constraints in real-world environments. While many approaches achieve high accuracy in experimental settings, practical adoption is still limited by connectivity issues, regional variability, and privacy concerns. The following subsections expand on these challenges and outline potential research directions, such as TinyML, LLMs, XAI, FL and TL, blockchain integration, and DT frameworks.

#### 3.6.1. TinyML in SM Forecasting

Real-time SM forecasting applications face several challenges in real-world environments. These include limited power, low-cost hardware, insufficient data, and processing delays. DL models like LSTMs, transformers, or hybrid methods usually need a lot of computing power and energy, which are hard to support in the field. Because of this, many real systems find it difficult to run these models continuously in real time.

Tiny Machine Learning (TinyML) [93] emerges as a promising solution. TinyML enables the deployment of lightweight, optimized ML models directly on low-power devices such as microcontrollers. By using techniques like pruning [94], quantization [95], and model distillation [96], TinyML significantly reduces model size and complexity, making it possible to run advanced models within the limited memory and processing capacity of these devices. This capability is particularly valuable in real-time SM forecasting, where quick decision-making—such as activating an irrigation system—requires low-latency predictions. As TinyML performs inference locally, without needing to send data to the cloud, it reduces dependency on continuous connectivity. While latency may not be a major concern in agricultural contexts, connectivity and reliable access to networks remain critical challenges, particularly for fields in remote or underdeveloped areas. By allowing local data processing, it also enhances data privacy and security, since sensitive environmental data does not need to be transmitted externally. In addition to these advantages, TinyML can be integrated with FL, enabling multiple distributed devices to collaboratively improve model accuracy without sharing raw data. This approach is useful in overcoming data scarcity and adapting models to local field conditions without compromising privacy.

In summary, TinyML directly addresses the core challenges of real-time SM applications by offering energy-efficient, fast, and privacy-aware solutions that are suitable for deployment in resource-constrained agricultural environments.

#### 3.6.2. LLMs and TinyLLMs for Soil Moisture Forecasting

Recent studies such as TimeGPT [64], ontology-driven LLM explanations for SM predictions [88], and domain-specific models like EnvGPT [89] highlight the potential of foundation models, but there are still many opportunities to strengthen their role in SM forecasting.

A first research direction is the development of domain-specific TinyLLMs that can operate on resource-constrained edge devices such as soil sensors, IoT hubs, or farm machinery. These compact models, fine-tuned on localized SM datasets, could provide real-time and offline predictions without reliance on cloud infrastructure. A second promising avenue is multimodal TinyLLMs, which combine textual reasoning with diverse data sources, including time-series soil sensor data, satellite imagery, and weather forecasts, thus offering more adaptive and holistic soil water forecasting systems. Future work should investigate TL and continual learning [97] strategies in TinyLLMs, enabling models trained in one climatic or soil condition to be incrementally adapted to another with minimal new data, directly addressing the data scarcity challenges in agriculture. Another important direction is the design of explainable TinyLLMs, capable of not only predicting SM but also providing user-friendly explanations linked to agronomic factors such as rainfall events, soil texture, and evapotranspiration. This would improve transparency and foster farmer trust in AI-driven recommendations.

Furthermore, integrating model compression and optimization techniques, such as pruning, quantization, and knowledge distillation, should be actively investigated to minimize the computational demands of TinyLLMs, thereby enabling their long-term deployment on low-power agricultural devices. Additionally, there is a pressing need to establish standardized benchmark datasets and evaluation protocols tailored to LLM and TinyLLM applications in soil moisture forecasting. This would facilitate fair comparisons, ensure reproducibility, and support consistent progress tracking across studies.

Collectively, these directions highlight a pathway that will be much explored in the future, where LLMs and TinyLLMs evolve from proof-of-concept demonstrations to practical, field-deployable tools for sustainable soil and water management, as well as precision agriculture.

#### 3.6.3. Model Interpretability and Trust

DL models often behave like black boxes, where the internal decision-making process is difficult to understand or explain. This lack of transparency can limit their adoption in agricultural settings, where farmers and stakeholders need to trust the system before relying on its predictions for critical tasks such as irrigation scheduling or drought management. Without interpretability, even highly accurate models may be viewed with skepticism, especially in real-world decision-making environments.

Some works, such as Wang et al. [65], have attempted to address this issue by incorporating explainability tools such as SHAPLY and t-SNE to visualize feature importance and data clustering. However, such approaches remain rare in SM forecasting, and most existing studies still report model accuracy without offering insights into how predictions are generated.

Future research should place greater emphasis on XAI techniques to build user trust and accountability. Possible directions include using SHAP values to quantify feature contributions, applying attention mechanisms to highlight which time steps or variables influence forecasts most strongly, and employing saliency analysis to visualize the sensitivity of predictions to input changes. These methods not only enhance transparency but also allow domain experts to validate whether the model is focusing on meaningful agronomic features. In the long run, integrating interpretability into SM forecasting models will be critical for bridging the gap between technical performance and practical acceptance in SA.

#### 3.6.4. Inconsistent Benchmarks and Evaluation Protocols

One major challenge in the current literature is the lack of consistency in datasets and evaluation protocols. Existing studies vary widely in terms of dataset duration (ranging from very short periods of 8 days to long-term records of 39 years), the types of input features considered (soil, weather, remote sensing, or crop-related variables), and the performance metrics used for model evaluation [22,26]. Such heterogeneity makes it difficult to compare results fairly across different works and often prevents meaningful conclusions about which models are truly more effective. For example, one study may report only RMSE and MAE, while another highlights MAPE and NSE, making direct performance comparisons unreliable.

This lack of standardization not only complicates cross-study benchmarking but also reduces the reproducibility and transparency of research findings. Without common benchmarks, it becomes unclear whether performance differences arise from the model architecture itself or from variations in data, preprocessing, and evaluation choices.

Future research should therefore prioritize the development of open benchmark datasets with common formats, well-documented sensor metadata, and predefined train-test splits to ensure comparability. In addition, adopting unified evaluation protocols, including a core set of performance metrics (e.g., MAE, RMSE, $R^{2}$, and MAPE) and standardized cross-validation strategies, will help establish fair baselines. Such efforts would enable the community to systematically compare methods, foster reproducibility, and accelerate progress in SM forecasting.

To formalize benchmarking and improve reproducibility in future soil moisture (SM) forecasting studies, a concise benchmark framework is proposed below. The goal is to ensure fair comparison across model architectures, data sources, and forecasting horizons.

- **Reference datasets:** International Soil Moisture Network (ISMN), ERA5, and NASA POWER are recommended as standard benchmark datasets that provide soil and meteorological variables with global coverage.
- **Input features:** Core predictors should include ST, RH, RL, AT, SR, and SEC, optionally extended with vegetation indices such as NDVI.
- **Forecast horizons:** Common prediction intervals depend on the case study, such as 1-day, 3-days, and 7-days, and even months-ahead forecasts are suggested to standardize temporal comparisons.
- **Data splitting:** Chronological training, validation, and testing splits (70–15–15) or temporal K-fold cross-validation schemes should be applied to avoid temporal data leakage.
- **Evaluation metrics:** MAE, RMSE, NRMSE, $R^{2}$, and NSE are recommended as the unified core metrics for model evaluation.
- **Reporting standards:** Studies should document sensor type, depth, site location, duration, and preprocessing procedures to enhance transparency and reproducibility.

Table 7 summarizes the proposed benchmark protocol for AI-based SM forecasting.

#### 3.6.5. Combine Federated and Transfer Learning

In SM forecasting, several studies have applied either TL or FL independently [64,74,77]. TL enables the adaptation of a pre-trained model, often trained on large-scale global datasets, to a smaller local dataset, thereby reducing data requirements and improving performance in regions where training data are scarce. FL, on the other hand, supports decentralized training by allowing multiple farms or devices to collaboratively train a model without sharing raw sensor data, thus preserving privacy and reducing communication bottlenecks.

Despite their individual benefits, the integration of TL and FL into a federated transfer learning (FTL) framework remains largely unexplored in the context of SM forecasting. Such an approach would allow each farm to locally fine-tune a powerful pre-trained model on its own sensor data, while still contributing model updates to a shared global model. This combination would maximize data efficiency, preserve privacy, and enhance model adaptability across diverse agro-ecological conditions.

Future work should therefore focus on designing and testing FTL frameworks tailored to agricultural applications. Key research directions include:

- Incorporating formal privacy guarantees (e.g., differential privacy parameters) to protect sensitive farm data.
- Evaluating energy and memory usage on low-power edge devices to ensure feasibility in real-world farm deployments.
- Testing resilience under real-world disruptions such as missing data, sensor failures, or adversarial scenarios to improve robustness.

Together, these steps will help transform early prototypes into trustworthy, scalable, and field-ready forecasting tools for SA.

#### 3.6.6. Blockchain for Data Security and Federated Learning Integrity

With the adoption of FL in SM forecasting, ensuring the security and trustworthiness of distributed model training becomes crucial. One potential solution is the integration of blockchain technology to create a secure, tamper-proof record of model updates across distributed nodes. Blockchain can help verify the authenticity of contributions from each farm or device in a federated network, detect anomalies or malicious updates, and manage participation in collaborative learning. Moreover, combining blockchain with FL can enable incentive-based data sharing, where farmers can receive tokens or credits for contributing updates without sharing raw data. In summary, research opportunities will be as follows:

- Design lightweight blockchain frameworks suitable for low-power agricultural devices.
- Integrate blockchain with FL to ensure transparency, model traceability, and auditability.
- Explore blockchain-based reward mechanisms for participatory sensing in smart farming.

#### 3.6.7. Dynamic Digital Twin Frameworks for Soil Systems

Digital Twins (DTs) are increasingly explored in agriculture, but their application to SM forecasting remains at a nascent stage. Existing works mainly focus on SM monitoring and classification rather than full forecasting. For example, Parewai and Köppen developed a DTs framework that integrates physically based rendering simulations with ML for SM state classification [98]. Similarly, a pasture management DTs model in South Africa included predictive components for soil and vegetation monitoring using regression and neural networks [99]. Broader surveys of agricultural DTs also highlight soil monitoring and irrigation management as key use cases, but often stop short of dynamic forecasting [100]. These approaches demonstrate the potential of DTs to link real-time sensor data with virtual soil models, but several limitations remain: (i) most DTs are static or physics-based, (ii) real-time adaptation to changing soil and weather conditions is limited, and (iii) forecasting horizons are either short or not considered.

AI-driven DTs [101,102] offer a way to overcome these issues. By embedding DL and hybrid models directly within the DT, it becomes possible to forecast future SM levels while continuously updating with real-time sensor inputs. Such DTs could adapt dynamically to seasonal variability, correct for sensor drift, and integrate heterogeneous data sources (soil, weather, etc). Moreover, combining DT frameworks with explainable AI would improve farmer trust, while lightweight AI models (TinyML or pruned DL architectures) would allow deployment on edge devices close to the field.

In summary, while existing DTs for SM focus mainly on monitoring and classification, the next step is to develop AI-driven, adaptive, and interpretable digital twins capable of accurate SM forecasting and real-time decision support in SA.

## 4. Conclusions

This survey, conducted following the PRISMA methodology, reviewed the landscape of AI-based soil moisture (SM) forecasting, focusing on the roles of traditional machine learning (ML), deep learning (DL), fine-tuning large language model (LLM) hybrid architectures, and modern learning paradigms such as transfer learning (TL) and federated learning (FL). While DL, fine-tuning LLM, and hybrid models have shown strong performance across a wide range of data features and geographies, challenges remain in achieving scalable, privacy-aware, and real-time forecasting suitable for deployment in smart agriculture (SA).

Overall, among the 68 reviewed studies, 32% employed ML models, 51% used DL architectures, 13% adopted hybrid frameworks, and 4% applied LLM-based approaches.

This review found that many studies still rely on centralized, data-rich settings, often without reporting key sensor metadata or addressing field-level constraints. TL offers a solution to limited data availability, while FL enables collaborative modeling without compromising data privacy. Recently, some studies have started adopting these paradigms to decentralize forecasting tasks across distributed agricultural settings. Both paradigms are still, however, in the early adoption stages within SM research.

Although this review provides a comprehensive synthesis of AI techniques applied to SM forecasting, it is not without limitations. The scope is largely restricted to approaches already implemented in the literature, and it does not extensively introduce novel AI methods from other domains that could be adapted to agriculture. This self-reflection highlights an opportunity for future surveys to go beyond existing applications and explore underutilized techniques with potential value for SM forecasting.

Future research in sensor-based soil moisture (SM) forecasting is expected to move towards the development of models that are not only accurate but also lightweight, interpretable, and adaptable to changing environmental conditions. In particular, the adoption of TinyML and TinyLLM frameworks represents a promising direction, enabling on-device intelligence and long-term deployment on low-power agricultural hardware. These approaches will likely be enhanced by the integration of explainable AI (XAI) methods, fostering greater transparency and trust in decision-making processes.

To support progress in this area, the establishment of standardized benchmark datasets, ideally open-access and covering multiple soil depths, will be essential for enabling reproducible evaluation and fair comparison across studies. Moreover, future efforts should focus on improving the reporting of metadata, implementing stronger privacy-preserving mechanisms, and promoting edge-level intelligence by deploying compact models directly on soil and weather sensors.

Finally, advancing explainable and domain-adaptive LLM architectures tailored for agricultural use cases could offer a new level of interpretability and support agronomic decision-making in a more informed and responsible manner. Collectively, these directions aim to make AI-based SM forecasting not only scientifically robust but also practical, inclusive, and sustainable in real-world agricultural contexts.

## Figures and Tables

**Table 2:** Traditional ML Models for Soil Moisture Forecasting.

*Papers*	*Publication * *Year*	*AI * *Models*	*Sensor* *Type*	*Sensor * *Depths*	*Data* *Features*	*Case Study* *(Duration, Place)*	*Evaluation * *Metrics*
[4]	*2017*	*DT, KNN,* *LR, SVM*	*FDR*	*10*	*SM, ST, ATT, PN,* *elevation, slope*	*1 year* *Romania*	*Accuracy* *MSE*
[17]	*2018*	*MLRs, RR,* *SVM,* *Weighted LR*	*Resistive* *base*	*-*	*SM, SE, ATT,* *HU, SR*	*1 week* *India*	*Accuracy,* *MSE, R* ^2^
[18]	*2019*	*Elastic-Net,* *GBM, MLRs,* *RF*	*Capacitance*	*-*	*SM, ST, HU,* *UV index, SR*	*37 days* *India*	*MSE,* *R* ^2^
[19]	*2020*	*LR,* *naive Bayes,* *PCA, SVM*	*FDR*	*-*	*SM, ATT, HU,* *VT*	*3 months* *India*	*-*
[20]	*2021*	*RF*	*FDR*	*10, 20,* *40, 5, 80*	*SM, WS, ATT,* *HU, RL, SR-V,* *indices, VT, CE*	*2 years* *Netherlands*	*Bias, R^2^, RMSE,* *unbiased RMSE*
[21]	*2022*	*SVM*	*NR*	*0–7*	*ST, SM, SE,* *ATT, HU, dew point,* *RL, LI, SRC, VI*	*1 year* *India*	*MSE,* *R* ^2^
[22]	*2021*	*SVM*	*NR*	*-*	*SM, ST, SEC,* *WD, WS, ATT,* *HU, RL, LI*	*1 year* *China*	*MSE,* *R* ^2^
[23]	*2022*	*AdaBoost, GBM,* *HBGB, LR,* *Lasso R, RF,* *RR, XGBoost*	*NR*	*15, 30,* *45*	*SM, ST, WBT,* *HU, PN,* *leaf wetness*	*2 years* *Turkey*	*MAE, R^2^,* *RMSE*
[24]	*2023*	*GBM,* *MLRs, RF*	*Resistive* *based*	*-*	*SM, ST, ATT,* *HU, SR*	*40 days* *India*	*MSE,* *R* ^2^
[25]	*2023*	*RF, SVM*	*NR*	*5*	*SM, WS, ATT,* *HU, RL*	*39 years* *Bangladesh*	*R, MAPE,* *R^2^, RMSE*
[26]	*2023*	*Bagging, Boosting,* *Max-Voting,* *RF, SVM, Stacking*	*FDR*	*-*	*SM, ST, soil pH,* *AP, HU, WS, ATT,* *PN, R, sun hours*	*3 years* *India*	*MAE, MSE,* *NSE, R^2^, RMSE*
[27]	*2025*	*RF*	*Sentek* *EnviroSCAN,* *rain gauge* *(RS-102D)*	*10,* *20*	*SM,* *RL*	*15 months* *Taiwan*	*MAE, MAPE,* *RMSE, R* ^2^
[28]	*2025*	*RF, GBRR,* *SVM, NN*	*In situ* *sensors*	*–*	*SM, SE, PN* *ST, soil texture*	*NR* *Sweden*	*RMSE, MAE* *R* ^2^
Soil Moisture (SM), Wind Speed (WS), Air Temperature (ATT), SR-Vegetation (SRV), Vegetation Type (VT), Crop Evapotranspiration (CE), Wind Direction (WD), Infiltration Rate (IR), Solar Radiation (SR), Rainfall (RL), Soil Evaporation (SE), Skin Reservoir Content (SRC), Vegetation Index (VI), Soil Electrical Conductivity (SEC), Light Intensity (LI), Atmospheric Pressure (AP), Vapor-Pressure Deficit (VPD), Average Temperature (AVT) Maximum Temperature (MXT), Minimum Temperature (MNT), Precipitation (PN), Humidity (HU)							

*Note*: “NR” denotes studies where the original publication did not specify the sensor model or depth but provided adequate soil or meteorological data for model training and evaluation. These were retained to ensure methodological completeness of AI-based forecasting approaches.

**Table 3:** DL Models for Soil Moisture Forecasting.

Papers	Publication Year	AI Models	Sensor Type	Sensor Depths	Data Features	Case Study Duration, Place	Evaluation Metrics
[29]	2017	ANNs	FDR	30	SM, WS, ATT, RL	6 months China	RMSE
[30]	2018	Dynamic ANNs	Cosmic ray	-	SM, soil type, WS, ATT, HU, PN, SR	4 years United Kingdom	MAE, R^2^, RMSE
[31]	2018	NN-MFPA, MLP-FFN, NN-PSO, NN-CS	NR	-	ST, ATT, HU	1 year Canada	RMSE
[32]	2018	ANNs, RF, SVM	NR	0–20, 20–40	Sandy proportion, SM, AP, WS, ATT, HU, PN, sun hours, UV index	2 years China	Accuracy, R^2^, RMSE
[33]	2018	MLRs, SVM, LSTM	Capacitance	5	SM	2 years+ United States	MSE, R^2^
[34]	2019	CNN	FDR	10, 20	ST, ATT, AP, HU, WS, PN-	4 years China	MAE, MSE, RMSE, R^2^
[35]	2020	KNN, ANNs, Polynomial R, SVM	NR	10, 30, 50	SM, ST, ATT	7 years Romania	Accuracy
[36]	2021	RF, SVM, ANNs,	NR	20, 30, 40	SM, soil type, ATT, drought, RL, age of plant	3 years France	MAE, R^2^, RMSE
[37]	2020	MLRs, ANNs, SVM	Capacitance, TDR	5	SM, RL	6 -3 months US–Australia	MSE, R^2^
[38]	2019	LSTM	Capacitance	10, 25, 50, 80	SM	1 year India	MAE, MAPE, MSE, RMSE
[39]	2022	RF, ARIMA, RNN, LSTM	NR	0–7, 7–28, 28–100, 100–289	SM, soil type, VPD, ATT, PN	9 years Serbia	MAE, MASE, SMAPE
[40]	2022	ANNs, PCA, LSTM	NR	-	SM, ST, WD, WS, ATT, HU, RL, LI, VT	3 years China	MAE, MAPE, RMSE
[41]	2021	ARIMA, Prophet, LSTM	FDR	10, 45, 80	SM	NR India	MAE, MSE, RMSE
[42]	2021	LSTM	FDR	-	SM, ST, ATT, HU	3 months China	MAPE, R^2^, RMSE
[43]	2021	ANNs	NR	-	ST, SEC, SM, ATT, HU, illuminance	2 months China	MAE, MSE
[44]	2022	DT, GNN, LR, LSTM, ANNs, RF	NR	20, 40, 60	SM, soil type, ATT, HU, WS, PN, SR	4 years Brazil	MAE, MAPE, R^2^, RMSE
[45]	2022	SVM, RF, Elman ANNs, RNN, LSTM	NR	10, 100, 200, 40	SE, SM	9 years Mongolia	MAPE, RMSE, MAE
[46]	2022	LSTM, SARIMA	Capacitance	-	SM, soil pH, ATT, HU, LI	18 days Sri Lanka	MAE, RMSE
[47]	2023	NAR, AEAR, ARIMA, SVM, Polynomial R, LSTM, ES	Capacitance, FDR	5, 10, 15, 20, 28, 30	SM	1 year United States	MAPE, max error
[48]	2023	LR, LSTM, Lasso R, modeling, SVM	TDR	120, 30, 7, 90	IR, SM, soil type, soil pH, ATT, VPD, HU, SR	1 year Australia	Accuracy
[78]	2022	SVM, ARIMA, LSTM	FDR	4	SM, ST, soil type	6 months China	MAE, RMSE
[49]	2022	ANNs, Probabilistic particle filter	Capacitance, FDR	30, 60	SM	4 years United States	NSE, RMSE mean biased error
[50]	2023	Logistic R, naive Bayes, RF, DT, KNN, SVM, ResNet50,	NR	-	SM, ST, WD, WS, ATT, HU, illuminance	United States	AUC, accuracy, F1, precision, recall
[51]	2023	ARIMA, LSTM	NR	10, 100, 40	SM, ATT, WS, PN	1 year China	MAE, MAPE, RMSE
[52]	2021	Deep Learning, MLRs, ANNs, SVM	NR	2, 25, 50	SM, ST, WS, ATT, RL	1 year India	MAPE, NSE, R^2^, RMSE
[53]	2023	LSTM	TDR	10, 20, 30	SM, ST, ATT, HU, PN	1 year South Korea	MSE, R^2^
[54]	2023	KNN, Lasso R, ANNs, RF, SVM	Capacitance	-	SM, ST, ATT, WS, HU, PN, RL, LI, vegetation indices	6 months Africa	R, MSE
[55]	2022	ANNs	Capacitance, FDR	30	SM	9 years United States	RMSE
[56]	2022	ARIMA, ANNs	NR	-	SM	8 days China	MAE, MAPE
[57]	2023	AOA, ELM, ANNs, SVM	FDR	-	SM, ST, SEC, ATT, HU	8 months China	MAE, MSE, R^2^
[58]	2022	BLSTM, LSTM, Encoder–Decoder	NR	10, 100, 200, 40	SE, SM, WS, runoff, vegetation indices	11 years Mongolia	MAE, MAPE, RMSE
[59]	2023	LSTM	Cosmic ray	-	SM, ST, AP, HU, PN, SR	6 years United Kingdom	MAE, MSE, R^2^, RMSE
[60]	2023	ARIMA, LSTM, ANNs, SARIMA, Sparrow Search	NR	10, 100, 200, 40	SE, SM, AP, visibility, WS, ATT, PN, RL, elevation	10 years Mongolia	MAE, R^2^, RMSE
[61]	2024	XGB, LightGBM, CatBoost , RF, kNN, LSTM,	TEROS 12, weather station	10	ST, EC, SR, ATT, AP, dew temperature	Winter: 2020–2021, 2021–2022: (Nov–Feb), USA	R^2^, MAE, MSE, RMSE, MAPE, NSE, U95, GPI
[62]	2025	AR, ARMA, ARIMA, MLR, NAR, NARX, MLPNN, LSTM, DRNN, CNN, MLPNN-Bagging, MLPNN-Boosting, MLPNN-AdaBoost	EC-TM probes	-	SM, daily PN	4 years USA	RMSE, MAE, GRI, PCC, NSE, MBE
[63]	2025	ConvLSTM, PredRNN, MIM, CubicRNN	ERA5 for SM, CN05.1 others	-	SM, PN, HU, WS, sunshine duration, AVT, MXT, MNT	1961–2020 (CN05.1), ERA5: 1979–now China	MAE, MSE, SSIM

**Table 4:** LLM-Based Models for Soil Moisture Forecasting.

Papers	Publication Year	AI Models	Sensor Type	Sensor Depths	Data Features	Case Study (Duration, Place)	Evaluation Metrics
[64]	2024	TimeGPT (Transformer-based)	Soil + Weather Sensors	Topsoil (0–30 cm)	SWP, PN, ST, Evapotranspiration, Soil Texture, Orchard Identity	2 Years, Belgium	MAE, RMSE (median + IQR 5-day horizon)
[88]	2025	Ontology-driven LLM	Soil Moisture Sensors + Ontology	NR	SM, Agronomic Ontology Features	NR (Ontology- based setup)	Accuracy, human-readable explanations
[89]	2025	EnvGPT (Fine-tuned LLM)	Environmental Corpora	NR	Soil & Water Management, Environmental Texts	NR (Domain- specific QA tasks)	QA accuracy, domain-specific benchmarks

**Table 5:** Hybrid Models for Soil Moisture Forecasting.

*Papers*	*Publication * *Year*	*AI * *Models*	*Sensor * *Type*	*Sensor * *Depths*	*Data * *Features*	*Case Study* *(Duration, Place)*	*Evaluation * *Metrics*
[65]	2023	RF, ELM, SVR 1D-CNN, LSTM, Transformer, CNN–LSTM, LSTM–CNN, CNN-with-LSTM, GAN-LSTM Feature Attention–LSTM, Temporal Attention–LSTM, Feature Temporal Attention–LSTM	ISMN, NASA POWER	0.05 m, 0.10 m, 0.20 m, 0.50 m, 1.00 m	SM, PN, ATT Longwave Radiation Shortwave Radiation WS, HU, ST	12 years (varies by site) 30 sites (Global)	R^2^, RMSE, SHAP, t-SNE visualization
[90]	2023	Encoder Decoder–LSTM	NR	5	SM, ST, ATT, PN, VT Longwave/Shortwave HU, Radiation, SR	18 years China	Bias, MAE, R^2^, unbiased RMSE
[66]	2023	BLSTM, LSTM, CNN-LSTM	Cosmic ray	-	SM, ATT, AP, WD, WS, Longwave/Shortwave HU, Radiation, SR	6 years United Kingdom	MAE, MSE, R^2^, RMSE
[68]	2024	Naive Bayes, VAR, ES, ARIMA, EGB, RF, N-BEATS, StemGNN	Capacitance	100, 15, 30, 5, 50, 60, 90	ST, SM, ATT, PN	2 years United States	MAE, MAPE, RMSE
[67]	2024	Attention–LSTM, GA, LSTM, ANNs, SVM	FDR	30	SM, ST, ATT, HU	9 years Canada	MAE, RMSE
[68]	2024	Naive Bayes, VAR, ES, ARIMA, EGB, RF, N-BEATS, StemGNN	Capacitance	100, 15, 30, 5, 50, 60, 90	ST, SM, ATT, PN	2 years United States	MAE, MAPE, RMSE
[69]	2023	Informer, PCA, Variational Mode Decomposition	NR	0–10	SM	16 years China	MAE, R^2^, RMSE
[70]	2024	Causal–Informer, Informer, LSTM, Autoformer, Reformer, ARIMA, RF–Informer, CORR–Informer, MI-Informer, NLGC–Informer, LGC–Informer	FLUXNET stations	Surface SM (5 or 10)	SM, ST, ATT, Short/Longwave Radiation, AP, PN, WS, VPD, CO_2_, Soil Heat Flux, Latent Heat Flux, Sensible Heat Flux	Up to 8 years (depends) 72 sites North America, Europe, Australia, etc.	RMSE, MAE, R^2^, CORR (Pearson)
[71]	2025	DT, RF, SVM, MLP, Hybrid Metablend Stacking Technique (HMST)	IMD and SM UN	5, 7.5, 15, 30, 45 cm	RL, SE, HU MXT, MNT, Sunshine Hours, Mean Temperature	6 years India	R^2^, MAE, MSE, RMSE
[72]	2025	XGB, RFR, SMR, CBR, BRF, ANFIS, ANFIS-GWO, RFR-CNN)	TDR Gravimetric method	10, 25, 50, 100	RA, SR, MXT, MNT, RH, ST, WS	NR United States	R^2^, MAE, MSE, RMSE
[73]	2025	GCCL (Graph Convolutional + ConvLSTM), ConvLSTM, CNN-LSTM, RF, SARIMAX	SMAP L4, ERA5-Land	5	RL Skin Temperature Surface Solar Radiation Wind Graph (Spatial-Temporal)	9 years 8 months China	RMSE, R^2^, mean bias, bias standard deviation

*Note*: “NR” denotes studies where the original publication did not specify the sensor model or depth but provided adequate soil or meteorological data for model training and evaluation. These were retained to ensure methodological completeness of AI-based forecasting approaches.

**Table 6:** Learning Paradigm for Soil Moisture Forecasting.

Papers	Publication Year	Learing Paradigm	AI Models	Sensor Type	Sensor Depths	Data Features	Case Study (Duration, Place)	Evaluation Process
[74]	2021	TL	CNN, LSTM, ConvLSTM	SMAP L4, ERA5-Land	Top 5 cm	SM, PN, ST	SMAP: 5 and ERA: 41 years China	R^2^, RMSE, bias
[75]	2024	TL	CNN, LSTM, MLP (ANN), Hybrid DL	SM CS650 reflectometers, weather station sensors	Horizon A and 2A (e.g., 5–92 cm, top to subsoil)	SM, ST, PN, ATT, HU, SR	3 years, 11 months 21 days Ecuador	MAE, RMSE, MSLE, NSE, KGE
[64]	2024	TL	TimeGPT, TFT, LSTM, VAR, ARIMA, Naive	SM + weather sensors	Topsoil (0–30 cm implied)	SWP, PN, SN, SE, RL Month Soil texture Pruning treatment Orchard identity	2 years Belgium	MAE, RMSE (median + IQR for 5-day forecasting horizon)
[76]	2024	FL	DNN, GB, RF (for feature selection), SHAP (XAI)	SM, DHT11 (temperature & humidity), LI, water level sensor	Multiple depths	SM, ST, HU Water Level LI	Around 1 month (continuous, every 10 s) India	RMSE, MAE, R^2^, training time, SHAP values
[77]	2024	FL	RF, NB, ANN (baseline)	Arduino-based SM sensors, water level sensors, DHT11 (temperature & HU), light sensors, RFID	Multiple depths	SM Water level ST, HU, LI	Short- term India	Accuracy, training loss, training time, communication overhead

**Table 7:** Proposed benchmark protocol summarizing standard datasets, features, metrics, and reporting rules for AI-based soil moisture forecasting.

Component	Recommended Standard	Purpose
Dataset	International Soil Moisture Network (ISMN), ERA5, NASA POWER	Ensure reproducibility and comparability across studies
Input Variables	SM/VWC, ST, RH, RL, AT, SR, SEC	Core environmental predictors for SM forecasting
Forecast Horizon	1-day, 3-days, and 7-days, even months ahead predictions	Maintain consistent temporal benchmarks
Data-Splitting Strategy	Chronological 70–15–15 (train–validation–test) or temporal K-fold cross-validation	Prevent data leakage and ensure temporal validity
Evaluation Metrics	MAE, RMSE, NRMSE, $R^{2}$, NSE	Unified accuracy and robustness assessment
Documentation Standards	Report sensor type, depth, site location, data duration, and preprocessing steps	Enhance transparency and reproducibility
